# 4-Amino-2,3,5-trimethyl­pyridine monohydrate

**DOI:** 10.1107/S1600536809016833

**Published:** 2009-05-20

**Authors:** Li-Yan Dai, Fu-Liang Zhang, Liang Shen, Ying-Qi Chen

**Affiliations:** a Department of Chemical Engineering, Zhejiang University, Hangzhou, People’s Republic of China; b College of Materials Chemistry and Chemical Engineering, Hangzhou Normal University, Hangzhou, People’s Republic of China

## Abstract

In the title compound, C_8_H_12_N_2_·H_2_O, four substituted pyridine mol­ecules alternate with four water mol­ecules, forming a large ring *via* O_water_—H⋯N_pyridine_ and N_amine_—H⋯O_water_ hydrogen bonding. Adjacent rings are connected *via* O_water_—H⋯O_water_ hydrogen-bonds, forming a three-dimensional network.

## Related literature

For pyridine-amine derivatives, see: Smith *et al.* (2005 ▶); Tsuzuki *et al.* (2005 ▶). For their role as chemical inter­mediates in the formation of diverse mol­ecules possessing biological activity, see: Birault *et al.* (2005 ▶); Gordon *et al.* (1996 ▶); Player *et al.* (2007 ▶). For related structures, see: Li *et al.* (2008 ▶); Lin *et al.* (2005 ▶); Xie *et al.* (2008 ▶); Yu *et al.* (2005 ▶); Zhou *et al.* (2005 ▶). For the extinction correction, see: Larson (1970 ▶).
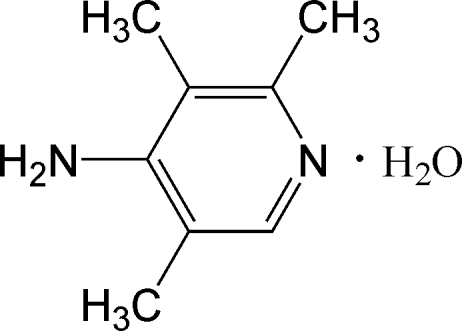


## Experimental

### 

#### Crystal data

C_8_H_12_N_2_·H_2_O
*M* *_r_* = 154.21
Tetragonal, 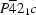

*a* = 19.5710 (9) Å
*c* = 4.8819 (2) Å
*V* = 1869.89 (14) Å^3^
*Z* = 8
Mo *K*α radiation
μ = 0.07 mm^−1^
*T* = 296 K
0.33 × 0.27 × 0.22 mm

#### Data collection

Rigaku R-AXIS RAPID diffractometer
Absorption correction: multi-scan (*ABSCOR*; Higashi, 1995 ▶) *T*_min_ = 0.967, *T*_max_ = 0.984
17243 measured reflections
1250 independent reflections
951 reflections with *F* ^2^ > 2.0σ(*F* ^2^)
*R*_int_ = 0.045

#### Refinement

*R*[*F* ^2^ > 2σ(*F* ^2^)] = 0.035
*wR*(*F* ^2^) = 0.088
*S* = 1.00
1250 reflections
101 parameters
H-atom parameters constrained
Δρ_max_ = 0.23 e Å^−3^
Δρ_min_ = −0.20 e Å^−3^

### 

Data collection: *PROCESS-AUTO* (Rigaku, 2007 ▶); cell refinement: *PROCESS-AUTO*; data reduction: *CrystalStructure* (Rigaku, 2007 ▶); program(s) used to solve structure: *SHELXS97* (Sheldrick, 2008 ▶); program(s) used to refine structure: *SHELXL97* (Sheldrick, 2008 ▶); molecular graphics: *ORTEP-3 for Windows* (Farrugia, 1997 ▶); software used to prepare material for publication: *WinGX* (Farrugia, 1999 ▶).

## Supplementary Material

Crystal structure: contains datablocks General, I. DOI: 10.1107/S1600536809016833/fl2246sup1.cif

Structure factors: contains datablocks I. DOI: 10.1107/S1600536809016833/fl2246Isup2.hkl

Additional supplementary materials:  crystallographic information; 3D view; checkCIF report

## Figures and Tables

**Table 1:** Hydrogen-bond geometry (Å, °)

*D*—H⋯*A*	*D*—H	H⋯*A*	*D*⋯*A*	*D*—H⋯*A*
O1—H101⋯N1	0.86	1.91	2.771 (2)	178
O1—H102⋯O1^i^	0.85	1.93	2.778 (2)	173
N2—H202⋯O1^ii^	0.87	2.17	3.009 (2)	161

Symmetry codes: (i) 
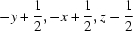
; (ii) 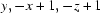.

**Fig. 1.**
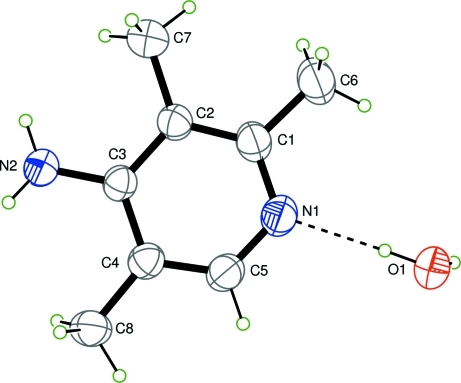
Molecular structure showing 40% probability displacement ellipsoids.

**Fig. 2.**
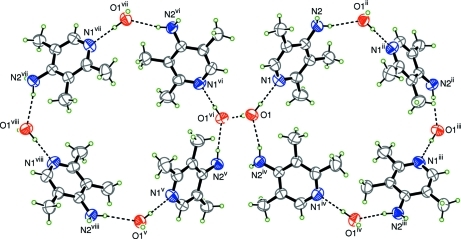
Hydrogen-bonding interactions.

## supplementary crystallographic information

### Comment

There is continuing interest in pyridin-amine derivatives due to their significant bioactivities (Smith *et al.*, 2005; Tsuzuki *et al.*, 2005) and their role as important chemical intermediates in the formation of diverse molecules possessing biological activities (Birault *et al.*, 2005; Gordon *et al.*, 1996; Player *et al.*, 2007). In general, compounds with amino groups can be used to prepare Schiff base ligands, which have played an important role in the development of coordination chemistry as they can readily form stable complexes with most metal ions (Lin *et al.*, 2005; Yu *et al.*, 2005; Zhou *et al.*, 2005). As part of our continuing investigation of such compounds, we report here the synthesis and crystal structure of a new pyridinamine derivative (Fig.1). Hydrogen-bonding interactions play an important role in the solid-state structure of this compound as they have in similar structures reported earlier (Li *et al.*, 2008; Xie *et al.*, 2008). As shown in Fig.2, four pyridine molecules and four water molecules are linked together alternatively to form a big ring *via* O_water_—*H*···N_pyridine_ and N_amine_—H···O_water_ hydrogen bonding (Table 1). Adjacent rings are connected to form a three-dimensional  network *via* O_water_—H···O_water _hydrogen-bonding. Channel can be seen within stacks of  the hydrogen bonded rings. The inner walls of the channels are occupied by the methyl groups and no solvent was found.

### Experimental

4-nitro-2,3,5-trimethylpyridine-N-oxide(18.2 g, 100 mmol), Raney nickel (25 g, 426 mmol) and 200 ml of ethanol were placed combined a three-necked flask. 80% Hydrazine hydrate(25 ml, 400 mmol) was added dropwise, maintaining the temperature under 35 degrees centigrade. The mixture was heated to reflux and 80% hydrazine hydrate was added dropwise continually. The catalyst was suction-filtered. Half of the ethanol was concentrated under vacuum. The residue was left at room temperature for 7 days giving some colorless needle shaped crystals suitable for data collection.

### Refinement

Friedel equivalents were merged. All H atoms were placed in calculated positions, with C—H = 0.93 or 0.96Å and N—H = 0.869 or 0.877 Å and included in the final cycles of refinement with a riding model, with *U*_iso_(H) = 1.2*U*_eq_(C,*N*,*O*).

### Crystal data

**Table:**

C_8_H_12_N_2_·H_2_O	*D*_x_ = 1.095 Mg m^−^^3^
*M**_r_* = 154.21	Mo *K*α radiation, λ = 0.71075 Å
Tetragonal, *P*42_1_*c*	Cell parameters from 10766 reflections
Hall symbol: P -4 2n	θ = 3.3–27.4°
*a* = 19.5710 (9) Å	µ = 0.07 mm^−^^1^
*c* = 4.8819 (2) Å	*T* = 296 K
*V* = 1869.89 (14) Å^3^	Chunk, colorless
*Z* = 8	0.33 × 0.27 × 0.22 mm
*F*(000) = 672.00	

### Data collection

**Table:**

Rigaku R-AXIS RAPID diffractometer	951 reflections with *F*^2^ > 2.0σ(*F*^2^)
Detector resolution: 10.00 pixels mm^-1^	*R*_int_ = 0.045
ω scans	θ_max_ = 27.4°
Absorption correction: multi-scan (*ABSCOR*; Higashi, 1995)	*h* = −25→25
*T*_min_ = 0.967, *T*_max_ = 0.984	*k* = −25→25
17243 measured reflections	*l* = −6→5
1250 independent reflections	

### Refinement

**Table:**

Refinement on *F*^2^	*w* = 1/[0.0001*F*_o_^2^ + 1.1100σ(*F*_o_^2^)]/(4*F*_o_^2^)
*R*[*F*^2^ > 2σ(*F*^2^)] = 0.035	(Δ/σ)_max_ < 0.001
*wR*(*F*^2^) = 0.088	Δρ_max_ = 0.23 e Å^−^^3^
*S* = 1.00	Δρ_min_ = −0.20 e Å^−^^3^
1250 reflections	Extinction correction: Larson (1970)
101 parameters	Extinction coefficient: 460 (64)
H-atom parameters constrained	

### Special details

**Table:**

Geometry. ENTER SPECIAL DETAILS OF THE MOLECULAR GEOMETRY
Refinement. Refinement using all reflections. The weighted *R*-factor (*wR*) and goodness of fit (*S*) are based on *F*^2^. *R*-factor (gt) are based on *F*. The threshold expression of *F*^2^ > 2.0 σ(*F*^2^) is used only for calculating *R*-factor (gt).

### Fractional atomic coordinates and isotropic or equivalent isotropic displacement parameters (Å^2^)

**Table:**

	*x*	*y*	*z*	*U*_iso_*/*U*_eq_	
O1	0.25888 (6)	0.28901 (6)	0.2514 (3)	0.0571 (4)	
N1	0.23938 (11)	0.42486 (10)	0.3906 (4)	0.0581 (6)	
N2	0.19626 (9)	0.61921 (9)	0.6961 (4)	0.0534 (6)	
C1	0.27642 (12)	0.47949 (12)	0.3109 (5)	0.0528 (7)	
C2	0.26474 (11)	0.54519 (11)	0.4090 (5)	0.0472 (6)	
C3	0.21206 (11)	0.55487 (11)	0.6011 (4)	0.0427 (6)	
C4	0.17323 (12)	0.49824 (12)	0.6859 (4)	0.0465 (6)	
C5	0.18992 (12)	0.43602 (12)	0.5762 (5)	0.0556 (8)	
C6	0.33136 (13)	0.46412 (12)	0.1049 (7)	0.0756 (9)	
C7	0.30672 (12)	0.60551 (12)	0.3136 (6)	0.0690 (9)	
C8	0.11654 (12)	0.50507 (12)	0.8924 (5)	0.0603 (7)	
H5	0.1649	0.3984	0.6350	0.067*	
H61	0.3753	0.4674	0.1913	0.091*	
H62	0.3251	0.4187	0.0345	0.091*	
H63	0.3288	0.4964	−0.0428	0.091*	
H71	0.3290	0.5943	0.1442	0.083*	
H72	0.2774	0.6442	0.2865	0.083*	
H73	0.3405	0.6163	0.4495	0.083*	
H81	0.1353	0.5184	1.0660	0.072*	
H82	0.0847	0.5391	0.8315	0.072*	
H83	0.0935	0.4620	0.9113	0.072*	
H101	0.2530	0.3317	0.2901	0.069*	
H102	0.2457	0.2771	0.0921	0.069*	
H201	0.1755	0.6229	0.8489	0.064*	
H202	0.2266	0.6511	0.6743	0.064*	

### Atomic displacement parameters (Å^2^)

**Table:**

	*U*^11^	*U*^22^	*U*^33^	*U*^12^	*U*^13^	*U*^23^
O1	0.0663 (10)	0.0465 (9)	0.0584 (10)	0.0063 (8)	−0.0044 (9)	−0.0047 (9)
N1	0.0701 (14)	0.0454 (11)	0.0587 (14)	0.0003 (10)	0.0039 (14)	−0.0022 (11)
N2	0.0624 (13)	0.0433 (11)	0.0545 (12)	−0.0029 (9)	0.0092 (11)	−0.0006 (10)
C1	0.0563 (16)	0.0549 (16)	0.0471 (14)	0.0083 (12)	0.0024 (13)	−0.0024 (13)
C2	0.0504 (14)	0.0454 (14)	0.0459 (13)	0.0003 (11)	0.0032 (14)	0.0021 (13)
C3	0.0472 (13)	0.0398 (12)	0.0411 (12)	0.0003 (10)	−0.0022 (12)	0.0001 (12)
C4	0.0507 (14)	0.0452 (13)	0.0435 (12)	−0.0002 (12)	−0.0022 (12)	0.0036 (13)
C5	0.0658 (17)	0.0454 (15)	0.0557 (15)	−0.0047 (12)	0.0015 (15)	0.0042 (14)
C6	0.086 (2)	0.0690 (19)	0.0717 (19)	0.0162 (16)	0.021 (2)	−0.0020 (18)
C7	0.0674 (17)	0.0627 (17)	0.077 (2)	−0.0054 (14)	0.0164 (17)	0.0013 (16)
C8	0.0640 (16)	0.0609 (15)	0.0562 (14)	−0.0072 (13)	0.0067 (15)	0.0060 (16)

### Geometric parameters (Å, °)

**Table:**

N1—C1	1.349 (3)	N2—H201	0.852
N1—C5	1.344 (3)	N2—H202	0.868
N2—C3	1.377 (2)	C5—H5	0.930
C1—C2	1.391 (3)	C6—H61	0.960
C1—C6	1.503 (3)	C6—H62	0.960
C2—C3	1.407 (3)	C6—H63	0.960
C2—C7	1.512 (3)	C7—H71	0.960
C3—C4	1.406 (3)	C7—H72	0.960
C4—C5	1.370 (3)	C7—H73	0.960
C4—C8	1.505 (3)	C8—H81	0.960
O1—H101	0.864	C8—H82	0.960
O1—H102	0.852	C8—H83	0.960
			
C1—N1—C5	116.9 (2)	C4—C5—H5	117.3
N1—C1—C2	123.0 (2)	C1—C6—H61	109.5
N1—C1—C6	114.8 (2)	C1—C6—H62	109.5
C2—C1—C6	122.2 (2)	C1—C6—H63	109.5
C1—C2—C3	118.3 (2)	H61—C6—H62	109.5
C1—C2—C7	121.8 (2)	H61—C6—H63	109.5
C3—C2—C7	119.9 (2)	H62—C6—H63	109.5
N2—C3—C2	120.82 (19)	C2—C7—H71	109.5
N2—C3—C4	120.0 (2)	C2—C7—H72	109.5
C2—C3—C4	119.1 (2)	C2—C7—H73	109.5
C3—C4—C5	117.2 (2)	H71—C7—H72	109.5
C3—C4—C8	121.7 (2)	H71—C7—H73	109.5
C5—C4—C8	121.1 (2)	H72—C7—H73	109.5
N1—C5—C4	125.4 (2)	C4—C8—H81	109.5
H101—O1—H102	115.0	C4—C8—H82	109.5
C3—N2—H201	118.6	C4—C8—H83	109.5
C3—N2—H202	117.5	H81—C8—H82	109.5
H201—N2—H202	111.9	H81—C8—H83	109.5
N1—C5—H5	117.3	H82—C8—H83	109.5
			
C1—N1—C5—C4	1.5 (3)	C7—C2—C3—N2	2.6 (3)
C5—N1—C1—C2	−0.9 (3)	C7—C2—C3—C4	179.6 (2)
C5—N1—C1—C6	179.6 (2)	N2—C3—C4—C5	177.6 (2)
N1—C1—C2—C3	0.3 (3)	N2—C3—C4—C8	−3.5 (3)
N1—C1—C2—C7	−179.4 (2)	C2—C3—C4—C5	0.6 (3)
C6—C1—C2—C3	179.8 (2)	C2—C3—C4—C8	179.5 (2)
C6—C1—C2—C7	0.1 (2)	C3—C4—C5—N1	−1.3 (3)
C1—C2—C3—N2	−177.1 (2)	C8—C4—C5—N1	179.8 (2)
C1—C2—C3—C4	−0.2 (3)		

### Hydrogen-bond geometry (Å, °)

**Table:**

*D*—H···*A*	*D*—H	H···*A*	*D*···*A*	*D*—H···*A*
O1—H101···N1	0.86	1.91	2.771 (2)	178
O1—H102···O1^i^	0.85	1.93	2.778 (2)	173
N2—H202···O1^ii^	0.87	2.17	3.009 (2)	161

Symmetry codes: (i) −*y*+1/2, −*x*+1/2, *z*−1/2; (ii) *y*, −*x*+1, −*z*+1.
